# Report of a Rare Case: A Maxillary First Molar with Seven Canals Confirmed with Cone-Beam Computed Tomography

**Published:** 2014-03-08

**Authors:** Rahul Kumar

**Affiliations:** a *Department of Conservative Dentistry and Endodontics, MGM Dental College and Hospital, Navi Mumbai, India*

**Keywords:** Cone-Beam Computed Tomography, Dental Operating Microscope, Maxillary First Molar, Root Canal Therapy, Tooth Abnormalities

## Abstract

**Introduction:** Coronal anatomic variations in permanent maxillary molars are unusual; conversely variations involving the number of root canals or number of roots are more common. **Methods and Materials:** This case report presents a successful nonsurgical endodontic therapy of left maxillary first molar with three roots and seven root canals. This unusual morphology was diagnosed using a dental operating microscope (DOM) and confirmed with the help of cone-beam computed tomography (CBCT) images. **Results:** CBCT axial images showed that both of the palatal and distobuccal roots had Vertucci type II canal pattern, whereas the mesiobuccal root canal showed a Sert and Bayirli’s type XV configuration. **Conclusion:** The use of a DOM and CBCT imaging in endodontically challenging cases can facilitate a better understanding of the complex root canal anatomy, which ultimately enables the clinician to explore the root canal system, and therefore treat it far more efficiently.

## Introduction

The variation of pulp cavity morphology, especially in multi-rooted teeth, is a constant challenge for diagnosis and successful endodontic therapy. Knowledge of the most common anatomic characteristics and their possible variations is fundamental because missing one canal can lead to endodontic treatment failure. Anatomic characteristics of permanent maxillary first molars are generally described as teeth with three [naming palatal (P), mesiobuccal (MB) and distobuccal(DB)] roots; each one containing a single canal, with the occurrence of a second mesiobuccal canal being common. The incidence of second mesiobuccal canal has been reported to be between 18% and 96.1% [1, 2,3].

Case reports with one [4], two[5], four [6], five [7] and six [8-12] root canals or with a C-shaped configuration of the canals [13] have also been reported earlier. Martinez-Berna and Ruiz-Badanelli reported a maxillary first molar with six root canals; three MB, two DB, and one P canal [14], whereas Maggiore *et al.* reported a maxillary first molar having six canals with two MB canals, three P canals, and one DB canal [15]. For the first time, Kottoor *et al.* reported maxillary first molars having seven [16] and eight [17] root canals. Alavi *et al.* [18] and Thomas *et al.* [19] reported the incidence of two canals in the DB root to be 1.90% and 4.30%, respectively. The incidence of two root canals in the P root of maxillary molars has been reported to be 2-5.1% [20]. Of the various comprehensive *in vitro *studies on the anatomy of maxillary first molars in the dental literature, only Baratto Filho *et al.* reported a maxillary first molar with three roots containing seven canals [21].

Recently advanced diagnostic aids like the dental operating microscope (DOM) and cone-beam computed tomography (CBCT) have helped the clinician to detect the hidden canals and to understand the variations in the root morphology. CBCT offers many advantages to the clinician due to its three dimensional reconstruction of root canal system and precise visualization of the radicular anatomy.

The present case is the second reported case in the literature which discusses the successful endodontic management of a maxillary first molar with three roots and seven root canals; three MB canals, two DB canals, and two P canals. This unusual morphology was confirmed with the help of CBCT and DOM.

**Figure 1.**
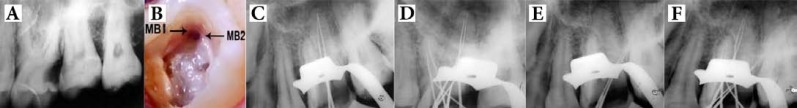
*A*) Preoperative radiograph of a maxillary first molar; *B*) Initial access opening; *C–F*) Eccentrically angulated radiographs to confirm the working length in the; *C*) Palatal root (P); *D*) Mesiobuccal (MB) root; and *E*) Distobuccal (DB) root. *F*) Access opening showing seven canal orifices

**Figure 2:**
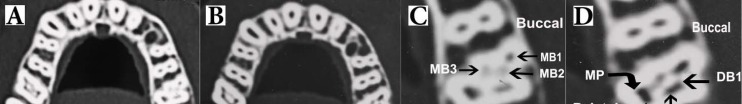
CBCT images of maxillary dental arc showing axial sections at the; *A)* Cervical and *B)* Apical level. Enlarged axial section of CBCT images at the; *C)* Cervical and *D)* Apical level, showing three roots and seven canals

**Figure 3:**
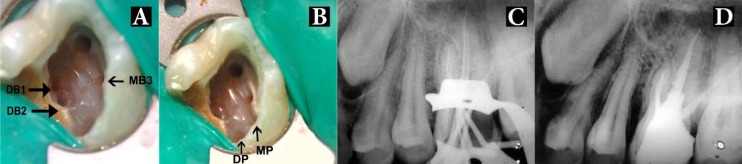
*A and B)* Access opening showing the seven root canal orifices; *C)* Master cone radiograph in eccentric angulation; *D)* Final radiograph

## Case Report

A 20-year old male patient referred to the department of Conservative Dentistry and Endodontics, with a chief complaint being spontaneous dull pain in posterior maxillary left region. The medical history was non contributory. Intraoral examination revealed a deep proximal carious lesion in maxillary left first molar with tenderness upon percussion. Clinical examination was suggestive of irreversible pulpitis in maxillary left first molar. A preoperative parallel radiograph revealed radiolucency in distal and occlusal areas of the crown, approaching the pulp space with widening of the periodontal ligament space surrounding the roots (Figure 1A). Initial radiographs of tooth suggested the presence of an unusual complex root anatomy with roots superimposed on each other. From the clinical and radiographic findings, a diagnosis of symptomatic apical periodontitis was made and endodontic treatment was offered to the patient.

The local anesthesia was performed using buccal infiltration injection of 2% lidocaine with 1:100,000 adrenalin (Lignox; Indoco Remedies, Mumbai, India). The tooth was isolated with rubber dam (Hygienic Dental Dam, Colténe Whaledent, Germany). After excavation of caries, a conventional endodontic access cavity was prepared and four canals were located (Figure 1B). During examination of the pulpal floor with DOM (Global Surgical Corporation, St. Louis, MO, USA), and an endodontic explorer (Star DG16) and a special ultrasonic tip (Start-X #3, Dentsply Maillefer, Ballaigues, Switzerland) two canal orifices were located in each of the three roots. After careful double checking, a third canal was located midway between the MB and P orifices. After exploring all the seven canals with #15 K-files (Kerr Manufacturing Co., Romulus, MI, USA), coronal enlargement of the root canals was done with ProTaper orifice shaper (Dentsply Maillefer, Ballaigues, Switzerland) to improve the straight line access (Figure 1B). The working length was determined with the help of an apex locator (Root ZX; Morita, Tokyo, Japan) and later confirmed with a radiograph. Individual intraoral periapical radiographs for the P (Figure 1C), MB (Figure 1D), and DB roots (Figure 1E) were taken to confirm the working lengths.

**Table 1:** Review of case reports of maxillary first molars with 6 and 7 canals

**Reference**	**No. of roots (canals)**	**Root (No. of canals)**	**Year**	**Type of study**
**Martınez-Bern and Ruiz-Badanelli (3 cases) [14]**	3 (6)	MB (3), DB (2), P (1)	1983	Clinical case-radiograph
**Bond ** ***et al*** **. [10]**	3 (6)	MB(2), DB (2), P (2)	1988	Clinical case-radiograph
**Maggiore ** ***et al*** **. [15]**	3 (6)	MB(2), DB(1), P (3)	2002	Clinical case-radiograph
**Adanir [11] **	4 (6)	MB (2), MP (1), DB (1), P (2)	2007	Clinical case-radiograph
**de Almeida-Gomes ** ***et al.*** ** [8] **	3 (6)	MB (2), DB (2), P (2)	2009	Clinical case-radiograph
**Karthikeyan and Mahalaxmi (4 cases) [24] **	3 (6)	MB (2), DB (2), P (2)	2010	Clinical case-Microscope and radiograph
**Albuquerque ** ***et al.*** ** (3 cases) [12]**	3 (6)	MB (2), DB (2), P (2)	2010	Clinical case-Microscope and radiograph
**Du Y. ** ***et al. *** **[13]**	3 (6)	MB (3), DB (1), P (2)	2011	Clinical case-Microscope and radiograph
**Kottoor ** ***et al.*** ** [16] **	3 (7)	MB (3), DB (2), P (2)	2010	Clinical case-CBCT and Microscope
**Present case**	3 (7)	MB (3), DB (2), P (2)	2013	Clinical case-CBCT and Microscope

Final working length radiograph was taken with an ideal angulation for confirmation of file position within the canals (Figure 1E). However, the radiographs did not clearly reveal the number and morphology of the root canal systems.

To ascertain this rare and complex root canal anatomy of the tooth, CBCT imaging was planned. Access cavity was sealed with IRM cement (Dentsply De Trey GmbH, Konstanz, Germany) and an informed consent was obtained from the patient and a multi-slice CBCT scan (CS9300, Carestream Health Inc, Rochester NY) of the maxilla was performed with a tube voltage of 100 kVp and a tube current of 8 mA. The involved tooth was focused, and the morphology was obtained in transverse, axial, and sagittal sections of 0.2 mm thicknesses. The CBCT images revealed that the tooth had three separated roots with seven distinct canals; three MB canals, two DB canals, and two P canals (Figure 2A-D). During careful examination of the CBCT scan, the contra lateral tooth also showed three separated roots and seven distinct root canals (Figure 2A).

## Discussion

Most endodontic literature, describe the human maxillary first molar with three roots and 3 to 4 root canals but great number of variations can occur in formation, number and shape of the roots [22]. Hence, changes in the operative procedures may be necessary. It is extremely important that endodontists use all the armamentarium at their disposal to locate and treat the entire root canal system [23]. Conventional radiographs, surgical operating microscope, ultrasonic instruments and CBCT are some of the useful diagnostic aids in endodontics [24,25]. The main disadvantage of a conventional film-based radiograph is that it provides a 2-dimensional image of a 3-dimensional object, resulting in superimposition of anatomical structures. As a newer diagnostic method, CBCT greatly facilitates the visualization of the internal root canal morphology [25]. One distinct advantage of CT scanning over the conventional radiograph is that it allows the operator to look at multiple slices of tooth roots and their root canal systems [26]. Various clinical studies have highlighted the role of CBCT as an objective analytic tool to ascertain root canal morphology [9,25].

Among the various case reports regarding the maxillary first molars in dental literature, only Kottoor *et al.* reported a maxillary first molar with three roots and seven root canals, which contained three MB, two DB, and two P canals [16]. Case reports of maxillary first molars presenting with six and seven canals are summarized in Table 1. The frequency of second canal in the MB root was reported to be 92.85% (based on *in vitro *results), 95.63% (based on clinical results), and 95.45% (based on CBCT results), whereas the corresponding figures for the DB root (DB2) were 1.15% (*in vitro*) and 3.75% (clinical) and for the P root the incidence of the second canal was 2.05% (*in vitro*), 0.62% (clinical), and 4.55% (CBCT) [16,17]. Most of the *in vitro* studies about the anatomy of MB root have not reported the presence of a third canal in the MB root [27,28]. Two of these studies have reported their incidence to be between 1.1% to 10% [27,29,30].

In the present case, CBCT scanning was used for a better understanding of the complex root canal anatomy .The use of CBCT in dentistry has increased dramatically in the past 2 decades [31,32]. With CBCT scans, it is possible to reconstruct overlapping structures at arbitrary intervals and thus the ability to resolve small subjects, is increased. Compared to conventional CT, they have drastically reduced scan time and effective dosages. In this case, CBCT axial images confirmed the presence of three roots and seven root canals, namely MB1, MB2, MB3, DB1, DB2, mesiopalatal (MP) and distopalatal (DP). The contra lateral tooth appeared to have the same root canal anatomy (Figure 2A and B). CBCT axial images also showed that both of the P and DB roots present a Vertucci type II canal configuration (*i.e.* two canal orifices that join together and exit as one apical foramen) [33], whereas the MB root canal showed a Sert and Bayirli’s type XV configuration (*i.e.*, MB1and MB2 joined at the middle third of the root and exited in one apical foramen) [34], whereas MB3 had a separate canal orifice and one apical foramen (Figure 2C and D). The MB2 canal is usually located more palatally and mesially from the MB1, but in this particular case the MB2 canal was located midway between the MB1 and DB1 canals (Figure 1B) and this peculiar location was confirmed in the CBCT axial images (Figure 2C and D). Thus, CBCT scanning was pivotal in the diagnosis of this unusual root canal system and towards its successful endodontic management.

## Conclusion

Knowledge of dental anatomy is fundamental for good endodontic practice. When root canal treatment is indicated, the clinician should be aware that both external and internal anatomy may be abnormal .The frequency of maxillary first molars with more than six root canals is very rare; however, each case should be clinically and radiographically investigated carefully to detect the anatomical anomaly. The present case report discussed the endodontic management of a maxillary first molar with seven canals and highlighted the role of DOM and CBCT scanning as objective analytic tools that enhance the overall success of endodontic therapy.

## References

[1] Kulid JC, Peters DD (1990). Incidence and configuration of canal systems in the mesiobuccal root of maxillary first and second molars. J Endod.

[2] Buhrley LJ, Barrows MJ, BeGole EA, Wenckus CS (2002). Effect of magnification on locating the MB2 canal in maxillary molars. J Endod.

[3] Purra AR, Mushtaq M, Robbani I, Farooq R (2013). Spiral computed tomographic evaluation and endodontic management of a mandibular second molar with four roots. A case report and literature review. Iran Endod J.

[4] Gopikrishna V, Bhargavi N, Kandaswamy D (2006). Endodontic management of a maxillary first molar with a single root and a single canal diagnosed with the aid of spiral CT: a case report. J Endod.

[5] Shakouie S, Mokhtari H, Ghasemi N, Gholizadeh S (2013). Two-rooted maxillary first molars with two canals: a case series. Iran Endod J.

[6] Christie WH, Peikoff MD, Fogel HM (1991). Maxillary molars with two palatal roots: a retrospective clinical study. J Endod.

[7] Barbizam JV, Ribeiro RG, Tanomaru Filho M (2004). Unusual anatomy of permanent maxillary molars. J Endod.

[8] de Almeida-Gomes F, Maniglia-Ferreira C, Carvalho de Sousa B, Alves dos Santos R (2009). Six root canals in maxillary first molar. Oral Surg Oral Med Oral Pathol Oral Radiol Endod.

[9] Dankner E, Friedman S, Stabholz A (1990). Bilateral C shape configuration in maxillary first molars. J Endod.

[10] Bond JL, Hartwell G, Portell FR (1988). Maxillary first molar with six canals. J Endod.

[11] Adanir N (2007). An unusual maxillary first molar with four roots and six canals: a case report. Aust Dent J.

[12] Albuquerque DV, Kottoor J, Dham S, Velmurugan N, Abarajithan M, Sudha R (2010). Endodontic management of maxillary permanent first molar with 6 root canals: 3 case reports. Oral Surg Oral Med Oral Pathol Oral Radiol Endod.

[13] Du Y, Soo I, Zhang CF (2011). A case report of six canals in a maxillary first molar. Chin J Dent Res.

[14] Martinez-Berna A, Ruiz-Badanelli P (1983). Maxillary first molars with six canals. J Endod.

[15] Maggiore F, Jou YT, Kim S (2002). A six-canal maxillary first molar: case report. Int Endod J.

[16] Kottoor J, Velmurugan N, Sudha R, Hemamalathi S (2010). Maxillary first molar with seven root canals diagnosed with cone-beam computed tomography scanning: a case report. J Endod.

[17] Kottoor J, Velmurugan N, Surendran S (2011). Endodontic management of a maxillary first molar with eight root canal systems evaluated using cone-beam computed tomography scanning: a case report. J Endod.

[18] Alavi AM, Opasanon A, Ng YL, Gulabivala K (2002). Root and canal morphology of Thai maxillary molars. Int Endod J.

[19] Thomas RP, Moule AJ, Bryant R (1993). Root canal morphology of maxillary permanent first molar teeth at various ages. Int Endod J.

[20] Stone LH, Stroner WF (1981). Maxillary molars demonstrating more than one palatal root canal. Oral Surg Oral Med Oral Pathol.

[21] Baratto Filho F, Zaitter S, Haragushiku GA, de Campos EA, Abuabara A, Correr GM (2009). Analysis of the internal anatomy of maxillary first molars by using different methods. J Endod.

[22] Ingle JI (Philadelphia). Endodontics.

[23] Vertucci FJ (2005). Root canal morphology and its relationship to endodontic procedures. Endodontic Topics.

[24] Karthikeyan K, Mahalaxmi S (2010). New nomenclature for extra canals based on four reported cases of maxillary first molars with six canals. J Endod.

[25] Kottoor J, Hemamalathi S, Sudha R, Velmurugan N (2010). Maxillary second molar with 5 roots and 5 canals evaluated using cone beam computerized tomography: a case report. Oral Surg Oral Med Oral Pathol Oral Radiol Endod.

[26] Patel S, Dawood A, Whaites E, Pitt Ford T (2009). New dimensions in endodontic imaging: part 1. Conventional and alternative radiographic systems. Int Endod J.

[27] Park JW, Lee JK, Ha BH, Choi JH, Perinpanayagam H (2009). Three-dimensional analysis of maxillary first molar mesiobuccal root canal configuration and curvature using micro–computed tomography. Oral Surg Oral Med Oral Pathol Oral Radiol Endod.

[28] Eder A, Kantor M, Nell A, Moser T, Gahleitner A, Schedle A, Sperr W (2006). Root canal system in the mesiobuccal root of the maxillary first molar: an in vitro comparison study of computed tomography and histology. Dentomaxillofac Radiol.

[29] Verma P, Love RM (2011). A Micro CT study of the mesiobuccal root canal morphology of the maxillary first molar tooth. Int Endod J.

[30] Degerness RA, Bowles WR (2010). Dimension, anatomy and morphology of the mesiobuccal root canal system in maxillary molars. J Endod.

[31] Ballal S, Sachdeva GS, Kandaswamy D (2007). Endodontic management of a fused mandibular second molar and paramolar with the aid of spiral computed tomography: a case report. J Endod.

[32] Reuben J, Velmurugan N, Kandaswamy D (2008). The Evaluation of Root Canal Morphology of the Mandibular First Molar in an Indian Population Using Spiral Computed Tomography Scan: An In Vitro Study. J Endod.

[33] Vertucci FJ (1984). Root canal anatomy of the human permanent teeth. Oral Surg Oral Med Oral Pathol.

[34] Sert S, Bayirli GS (2004). Evaluation of the root canal configurations of the mandibular and maxillary permanent teeth by gender in the Turkish population. J Endod.

